# Trends in orthodontic scientific contributions: An evaluation based on the American Association of Orthodontists annual sessions

**DOI:** 10.1371/journal.pone.0324810

**Published:** 2025-05-20

**Authors:** Ignacio García-Espona, Ahmed Amine Kanine-Ait-Zalim, José Antonio Alarcón, Cristina García-Espona, Eugenia García-Espona

**Affiliations:** 1 Department of Stomatology, Section of Orthodontics, School of Dentistry, Campus Universitario de Cartuja s/n, University of Granada, Granada, Spain; 2 President of the Spanish Association of Orthodontists (AESOR), Madrid, Spain; University of the Witwatersrand Johannesburg Faculty of Health Sciences, SOUTH AFRICA

## Abstract

Scientific contributions (lectures and posters) to the American Association of Orthodontists (AAO) annual sessions from 2013 to 2023 were investigated with the aims of analysing the contributions of each country and their efficiency, presentation trends, and gender differences during these years as well as the most frequent topics and their evolution. Official data were requested from and provided by the AAO secretary. The year and type of presentation; the name, country and gender of the first author; and the full title of the presentation were considered. In addition, six national indicators that could determine the quantity and quality of scientific production were obtained from the Our World in Data website with regard to the countries that made the greatest contributions to the AAO annual sessions. The USA featured the largest number of lecturers (69.44%), while the presentations of posters were more balanced among the 4 countries that exhibited the highest levels of production (i.e., Brazil, the USA, Mexico and South Korea). Brazil was the main country to perform above expectations. The COVID-19 pandemic resulted in a significant reduction in the number of poster presentations. The male/female ratio was close to 3:1 in terms of lectures and close to 1:1 in terms of posters. In 2023, women presented more posters than did men. The terms clear/aligners and digital were strongly present, and the terms maxillary, adults, and expansion were used increasingly frequently, while the use of the terms brackets or cephalometry decreased. American lecturers included terms that differentiated them from lecturers in other countries. The nationalities of lecturers are not closely related to those of posters, particularly with regard to the USA, Brazil, Canada, Mexico and Turkey. Research spending and economic level are the most significant factors with respect to the type and number of a country’s contributions. Concerning gender, a clear imbalance in favour of men persists among lecturers. Increased distance from the USA makes it more difficult for women to serve as lecturers. An emergent paradigm shift in current topics towards a focus on the terms clear/aligners and digital in lectures is evident.

## Introduction

The field of orthodontics is undergoing continuous evolution, which is primarily leading to increasingly rapid changes in our approach to diagnostics and therapy. It is important to validate these changes in an objective way as well as to reevaluate them over time. Accordingly, it is necessary to perform a multinational study that includes both clinical and research fields in such an evaluation to develop a wider approach to the new situation in the field of orthodontics.

In the context of such a hybrid approach, the contributions to the annual conference of the American Association of Orthodontists (AAO) could be used as representative indicators of trends in orthodontics. The AAO is the world’s longest-standing dental specialty organization and represents 18,920 orthodontist members throughout the USA, Canada and abroad. At present, 3,048 members (16.1%) of the association are international members or international students [1,2]. This diverse membership reflects the global reach of the AAO and its importance as a platform for sharing orthodontic knowledge and experience at the international level.

Clear differences among countries have been reported in terms of their contributions to general health [3], dentistry [4], and orthodontics [5] research. These variations are related to numerous diverse national economic and research indicators, such as research and development (R&D) manpower, spending and productivity, investment in research infrastructure, the accessibility of research funds, the share of research performed by the academic sector and funded by the private sector, the number and quality of scientific journals, publications and patents, among others [6]. These variations have also been related to more specific national indicators in this area, such as the number of students and professionals, the degree of technological or educational specialization and participation in scientific conferences, among others.

Additionally, authors of publications in the field of dentistry have also been shown to differ notably in terms of gender [7,8] and in orthodontics [9,10]. The lower presence and relevance of women in the medical research field is a constant issue that has also been reported in science, technology, engineering, mathematics and medicine (STEMM) fields as well as in healthcare in general [11–14]; furthermore, this gap extends beyond the level of publications to encompass the boards of directors of scientific societies [15,16], professional associations [16,17], and editorial committees of scientific dental journals [18,19], as well as faculty members in dental schools, particularly those occupying the highest academic positions [10]. Easily detected macroaggressions coexist with subtle discriminations, and it has been suggested that the phenomenon of gender bias may be underestimated, given that articles on gender bias are funded less often and published in journals with a lower Impact Factor than articles on comparable instances of social discrimination [20].

Finally, the topic of scientific presentations has varied substantially over time as the lines of development of orthodontics have changed [5,21–23]. The emergence of new materials, techniques, diagnostic and therapeutic tools and even treatment philosophies has led to the evolution of this discipline, i.e., orthodontics, which has exhibited a continuous tendency to change.

In this study, we investigated scientific contributions (lectures and posters) to the AAO annual sessions from 2013 to 2023 with the goals of analysing the contributions of each country and their efficiency with respect to certain indicators, examining trends in the evolution of presentations, investigating differences by gender over the years, and identify the most frequently discussed topics and their evolution.

## Materials and methods

### Design and setting

This study employs an observational, descriptive, cross-sectional design to investigate secondary data drawn from scientific presentations made at the annual sessions of the AAO from 2013 to 2023. The analysis of the past decade, which was sufficient to support inferences regarding trends, was expanded to encompass an additional year due to the atypicality of the year 2020, which was characterized by the COVID-19 pandemic.

Original official data from all lectures and posters presented at the AAO’s annual sessions (2013–2023, inclusive) were requested from the AAO secretary, who kindly provided them to us. These data are not easy to obtain because they are not grouped annually and are not always updated in the programmes at the end of the conferences due to late cancellations.

### Measures and variables

The main variables included in this study are associated with each type of presentation (lecture or poster) in the AAO’s annual sessions. The year and type of presentation; the name, country and gender of the first author; and the full title of the presentation were considered. An exhaustive post hoc internet search was conducted to confirm the countries and genders of some authors.

In addition to these data, six national indicators that could determine the quantity and quality of scientific production were obtained from the Our World in Data website [24] for countries that made the most contributions to the AAO annual sessions: population, gross domestic product (GDP), researchers per million inhabitants, total number of researchers, percentage of research spending and dentists per ten thousand inhabitants. These variables were included for two main reasons: they represented a good benchmark of the country’s research and economic capacity, and they were the only localized data available for all 24 top contributing countries. A seventh indicator, i.e., the distance between each country and the USA, was added to measure the possible difficulty of attending the congress on the part of researchers from that country.

These national indicators were associated with the quantity of each type of production categorized by gender as well as with a new variable (“Weighted Scientific Production”), in which context the production of lecturers was assigned a value triple that assigned to posters.

### Data analyses

First, the scientific production of each country was determined by conducting a descriptive analysis of percentages and frequencies. The comparative percentages of lecture and poster production were also analysed by reference to the production ratio of each country.

Second, we considered the linear relationships between the national indicator variables and the country’s contributions to the annual sessions by reference to the Pearson correlation coefficient [25]. Furthermore, to measure the efficiency of the presentations of each country, we developed a “national global indicator” (NGI), which involved adding the normalized values of the national indicators by transforming them to values associated with a normal distribution with a mean of 0 and deviation of 1. To create the NGI, seven variables were chosen that we considered relevant for each country and that were available in open data: Population, GDP, Researchers per Million Inhabitants, Total Number of Researchers, Percentage of Research Expenditure and Dentists per ten thousand Inhabitants. The seventh indicator was also taken as the distance to the US for each country, as a measure of the potential difficulty of attending congress. With these data, the NGI was created by normalizing the variables (i.e., transforming them into values belonging to a normal distribution of mean 0 and deviation 1 in a typing process) and adding their values to obtain the global indicator.

We omitted the number of researchers from this NGI because of its high correlation with the number of researchers per million. In addition, the USA was excluded from these analyses because it is the organizing country and accounts for nearly 70% of lecturers.

By reference to this NGI, we established a ranking based on the score, which we utilized as the position that each country should have exhibited in terms of its contributions to the AAO’s sessions. We compared countries’ positions based on this global indicator with their real positions with regard to each type of production to determine which countries outperformed and underperformed their expected scientific production.

Third, we reviewed the differences by gender by conducting a comparative analysis of the values for each country between men and women, including by comparing percentages and frequencies with regard to both lectures and posters. This analysis was conducted in conjunction with a global analysis of the trends observed in each country over the years 2013–2023.

Finally, we conducted a qualitative analysis, which specifically involved an outline of a thematic analysis [26] of the titles of the lectures and posters, with the goal of identifying possible differences in the topics under analysis over the years as well as possible differences among countries or between different presentation formats. This thematic analysis was based on the principles of text mining and sought to identify the most frequently used terms, their appearance and their relationships with other terms. This analysis was conducted with the assistance of using two basic statistical packages, i.e., tm and wordcloud [27,28], based on R software.

To accomplish this goal, before obtaining the thematic clouds, it was necessary to eliminate connecting words; to exclude very common or nonspecific terms that do not provide much additional information (orthodontic/s, patient/s, tooth/teeth, class, practice, clinical, management, treatment/treating, use/using, improve, correction and effects); and to link similar terms as well as plural and singular forms. However, these terms were not eliminated in the clouds used to facilitate comparisons between periods, presentation formats and countries with the goal of observing their variation over time.

## Results

### Contribution of each country and efficiency

**Fig 1:**
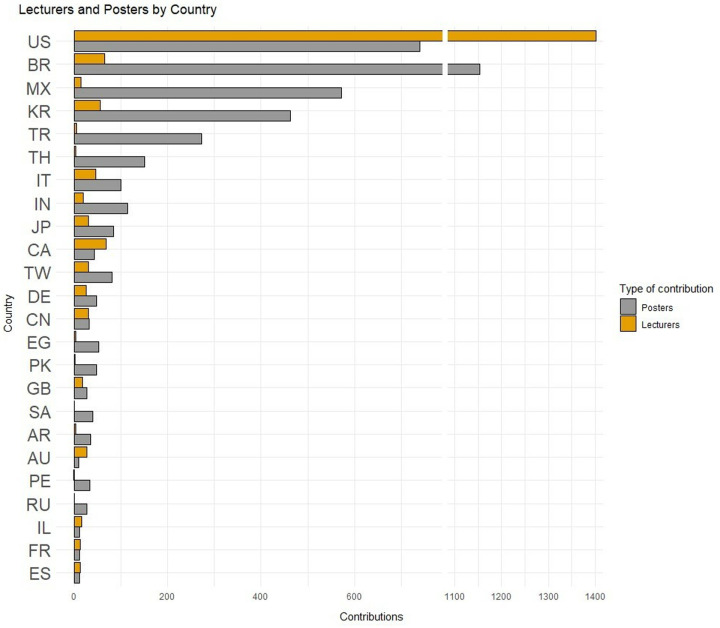
Lectures and posters by country (2013-2023) [Only countries featuring at least 25 contributions] (Abbreviations: US: United States of America, BR: Brazil, MX: Mexico, KR: South Korea, TR: Turkey, TH: Thailand, IT: Italy, IN: India, JP: Japan, CA: Canada, TW: Taiwan, DE: Germany, CN: China, EG: Egypt, PK: Pakistan, GB: United Kingdom, SA: Saudi Arabia, AR: Argentina, AU: Australia, PE: Peru, RU: Russia, IL: Israel, FR: France, ES: Spain).

The results regarding lectures and posters by country were added from 2013 to 2023, and the analysis was limited to the 24 countries that featured at least 25 contributions between lectures and posters (Fig 1). The USA was the country that produced the largest number of lecturers, accounting for nearly 70% of the total number, followed far behind by Canada and Brazil, which accounted for approximately 3% of lecturers. As many as 71 different countries from the five main continents presented posters during the period under investigation, although the three countries that featured the highest participation rates were located in North or South America (i.e., Brazil, the USA and Mexico).The presentation of posters was more balanced among the 4 countries that exhibited the highest levels of production (Brazil, the USA, Mexico and South Korea), although Brazil was the most notable, accounting for more than one quarter of the total number of posters and exhibiting higher levels of production than the USA.

Among the 10 countries that exhibited the highest levels of scientific production in both formats (Table 1), contributions in poster format were more frequent than presentations by lecturers, with the exceptions of the USA, Canada and Australia. On the other hand, Thailand, Turkey, Mexico and Brazil exhibited very high poster/lecture ratios.

**Table 1:** Top ten countries in terms of lecturers (left) and posters (right).

Top 10 Lecturers	Quantity	% Total Lectures)	Lecture/Poster Ratio	Top 10 Posters	Quantity	% Total Posters)	Poster/Lecture Ratio
USA	1402	69.44%	1.89	Brazil	1154	26.79%	17.48
Canada	69	3.42%	1.60	USA	740	17.18%	0.53
Brazil	66	3.27%	0.06	Mexico	572	13.28%	38.13
South Korea	56	2.77%	0.12	South Korea	462	10.73%	8.25
Italy	47	2.33%	0.47	Turkey	273	6.33%	45.51
Japan	31	1.54%	0.36	Thailand	151	3.51%	50.33
China	30	1.48%	0.37	India	115	2.67%	6.05
Taiwan	30	1.48%	0.94	Italy	100	2.32%	2.13
Australia	28	1.39%	2.80	Japan	85	1.97%	2.74
Germany	26	1.29%	0.54	Taiwan	81	1.88%	2.7

**Fig 2:**
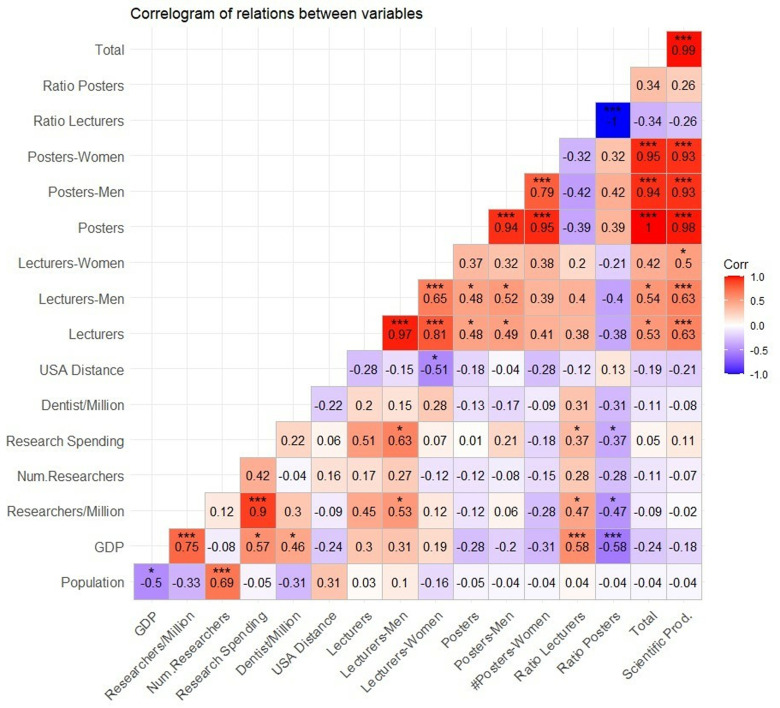
Correlogram of the relationships among the variables.

A correlogram (Fig 2) reveals the correlations among the variables associated with scientific contributions to the annual sessions (lectures and posters divided between men and women), the seven indicators mentioned above, the “Weighted Scientific Production” variable, and the distance between the capitals of the countries. The USA was excluded from this correlogram because it was considered to constitute an outlier, and Taiwan was excluded because we did not have access to data concerning researchers, dentists, and research spending for this country.

The efficiency analysis of the countries is presented in Table 2, in which the countries in the first column are ordered on the basis of the value of our NGI. Brazil was most notable in terms of its performance above expectations with regard to both lectures and posters, followed by Mexico. India, Italy, China and Australia were also notable in terms of lectures, while Turkey, Thailand, India, Egypt and Pakistan were notable in terms of posters. The positions of Asian countries such as South Korea and Japan were more or less in line with expectations, whereas some European countries such as Germany, France or Spain and the UK were less efficient than expected.

**Table 2:** Efficiency analysis of various countries [left column: countries ordered according to our national global indicator; central column: countries ordered according to the number of lectures they produced; right column: countries ordered according to the number of posters they produced].

Countries	Countries (Lectures)	Countries (Posters)
1 - Germany	1 - Canada ▲	1 - Brazil ▲▲
2 - South Korea	2 - Brazil ▲▲	2 - Mexico ▲▲
3 – Israel	3 - South Korea ▬	3 - South Korea ▬
4 - Canada	4 - Italy ▲▲	4 - Turkey ▲▲
5 – France	5 - Japan ▬	5 - Thailand ▲▲
6 – Japan	6 - China ▲	6 - India ▲▲
7 – UK	7 - Australia ▲▲	7 - Italy ▲
8 – Spain	8 - Germany ▼▼	8 - Japan ▼
9 – Italy	9 - India ▲▲	9 - Egypt ▲▲
10 – China	10 - UK ▼	10 - Germany ▼▼
11 - Argentina	11 - Israel ▼▼	11 - Pakistan ▲▲
12 - Australia	12 - Mexico ▲▲	12 - Canada ▼▼
13 - Russia	13 - France ▼▼	13 - Saudi Arabia ▲
14 – Brazil	14 - Spain ▼▼	14 - Argentina ▼
15 - Turkey	15 - Turkey ▬	15 - Peru ▲
16 - Saudi Arabia	16 - Egypt ▲	16 - China ▼▼
17 – India	17 – Argentina▼▼	17 - UK ▼▼
18 - Mexico	18 - Thailand ▲	18 - Russia ▼▼
19 – Peru	19 - Pakistan ▲	19 - France ▼▼
20 - Egypt	20 - Peru ▬	20 - Spain ▼▼
21 - Thailand	21 - Russia ▼▼	21 - Israel ▼▼
22 - Pakistan	22 - Saudi Arabia ▼▼	22 - Australia ▼▼

▲ Countries performing above expectations

▬ Countries exhibiting the expected level of performance

▼ Countries performing less efficiently than expected

### Trends and gender differences

**Fig 3:**
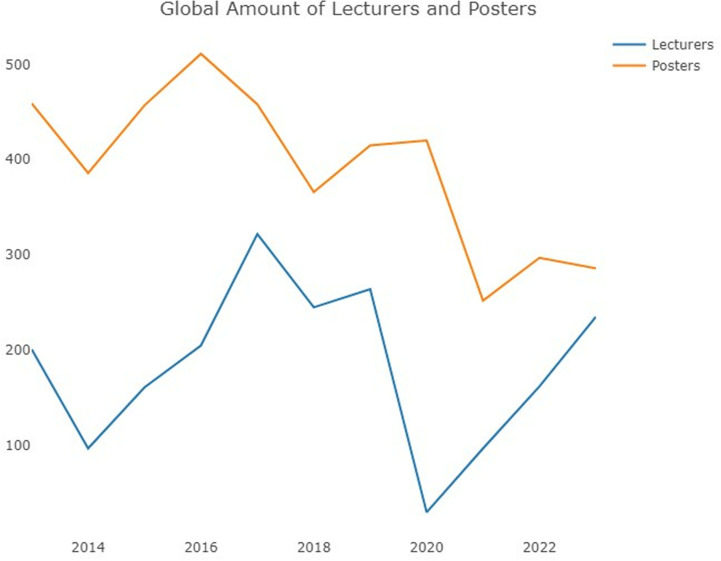
Global number of lectures and posters (2013-2023).

In terms of *year*, the total number of lectures and posters from every country (Fig 3) reveals that, until 2016, the number of posters was double that of lectures as a general rule. The pandemic led to a sharp and enormous decrease in lectures (which were shifted to an online format), and lectures exhibited a much greater decrease in 2020 than did posters. Thereafter, this number increased once again, but differences were observed between posters and lectures in this context: the numbers of both types of contributions converged in the past two years, which was mainly due to the decrease in the number of posters, whereas the number of lectures exhibited a quicker recovery.

**Fig 4:**
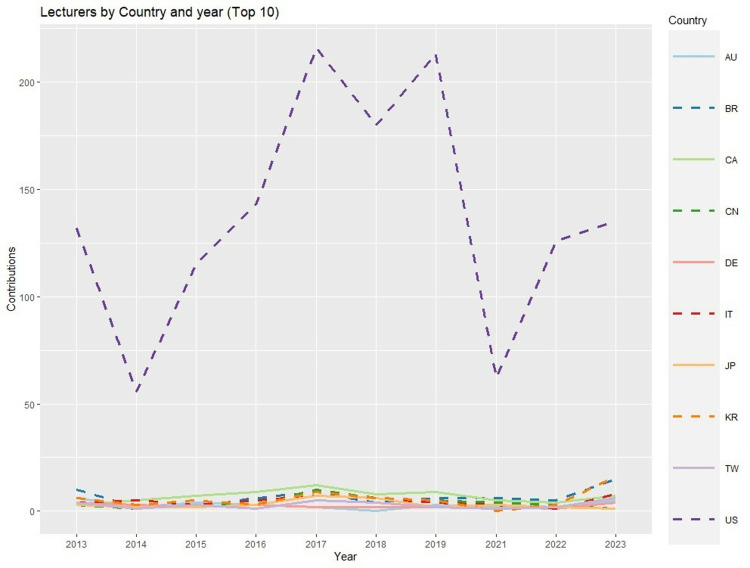
Lectures by country and year [Top 10 countries] (Abbreviations: AU: Australia, BR: Brazil, CA: Canada, CN: China, DE: Germany, IT: Italy, JP: Japan, KR: South Korea, TW: Taiwan, US: United States of America).

**Fig 5:**
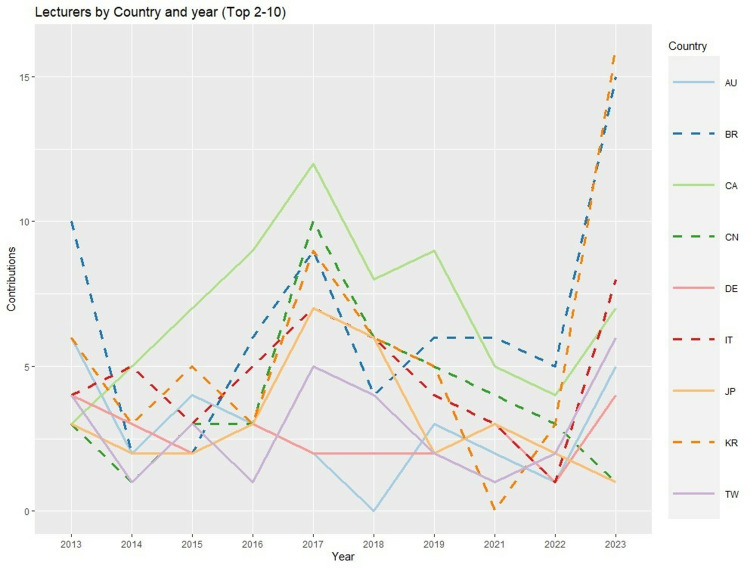
Lectures by country and year [Top 2-10 countries] (Abbreviations: AU: Australia, BR: Brazil, CA: Canada, CN: China, DE: Germany, IT: Italy, JP: Japan, KR: South Korea, TW: Taiwan).

With respect to year and country, the predominance of the USA in terms of lecturers (Fig 4) was so overwhelming that it was necessary to present the remaining top 10 countries in a distinct figure (Fig 5). In 2023, the number of lecturers from other countries increased (especially with regard to KR, BR, and IT), although the difference from the organizing country was still overwhelming. Canada, Japan, and China were notable in a negative way, as the number of lecturers from each of these countries have been decreasing in recent years.

**Fig 6:**
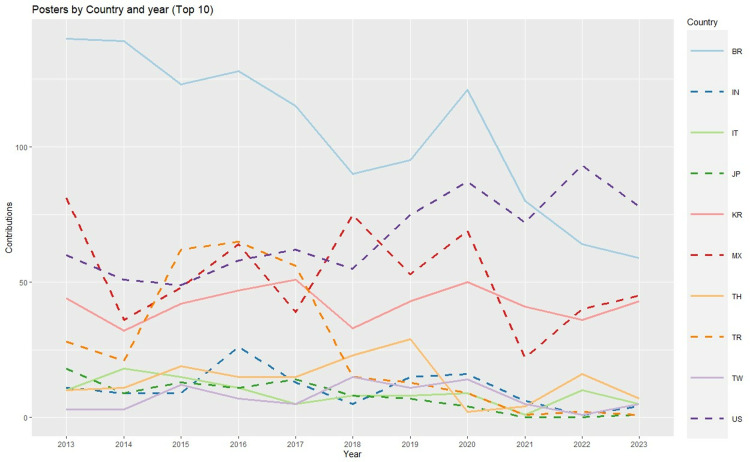
Posters by country and year [Top 10 countries] (Abbreviations: AU: Australia, BR: Brazil, CA: Canada, CN: China, DE: Germany, IT: Italy, JP: Japan, KR: South Korea, TW: Taiwan, US: United States of America).

With regard to posters (Fig 6), Brazil, the most active country in terms of presentations, progressively reduced its contributions since 2013 (with the exception of 2019–20); accordingly, the USA has overtaken Brazil in the past two years. The other countries maintained relatively stable temporal trends over time, with the exceptions of Turkey, which exhibited a large presence between 2015 and 2017 but reduced its presence significantly in recent years, and Mexico, which exhibited more irregular contributions.

**Fig 7:**
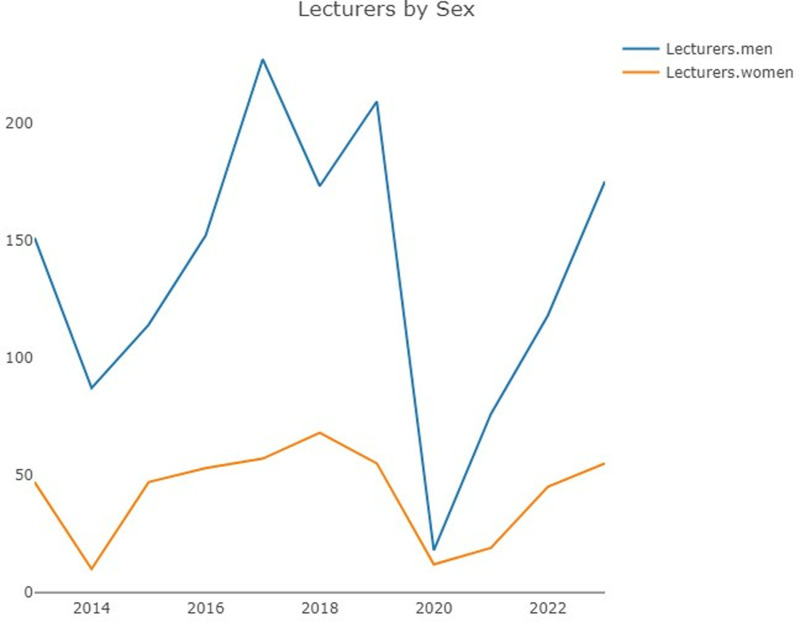
Lecturers by gender.

**Fig 8:**
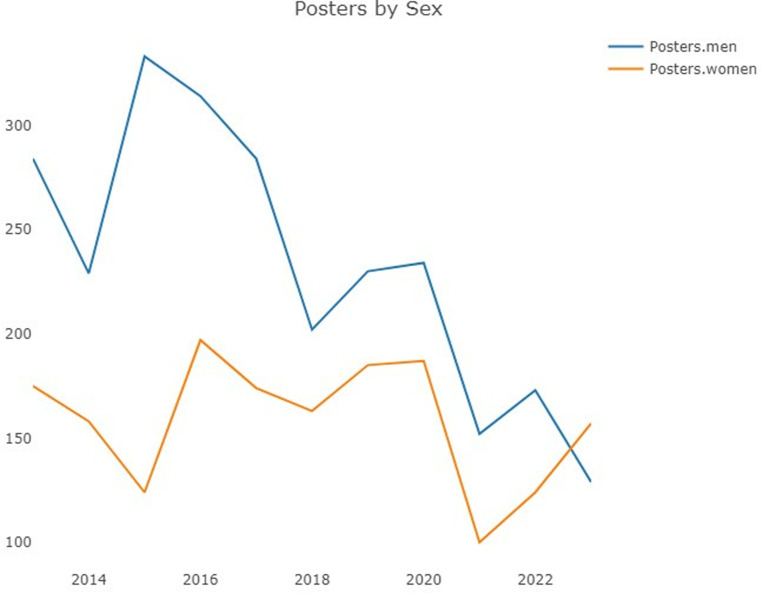
Posters by gender.

In terms of *gender*, the total number of lecturers (Fig 7) was characterized by a male/female ratio close to 3:1, and the difference, which clearly decreased in 2020, increased once again in 2023. However, with respect to posters (Fig 8), the male/female ratio is more balanced, nearing 1:1 in the past five years of the analysis until 2023, when women were observed to have a greater presence than men for the first time.

**Fig 9:**
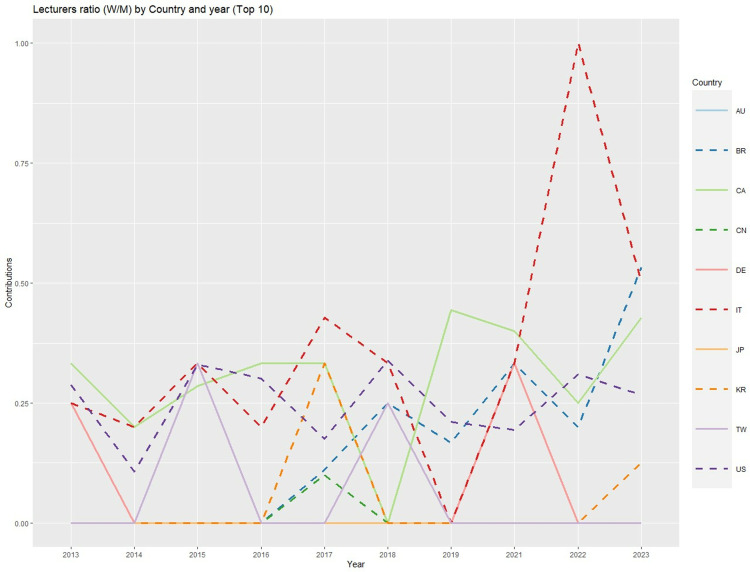
Lecturer ratios (women/men) by country and year [Top 10 countries] (Abbreviations: AU: Australia, BR: Brazil, CA: Canada, CN: China, DE: Germany, IT: Italy, JP: Japan, KR: South Korea, TW: Taiwan, US: United States of America).

Among the top 10 countries in terms of lecturers (Fig 9), the USA has exhibited similarly low proportions of women over the years, thus indicating that the total number of women lecturers is always lower, as the USA features the highest proportion of lecturers. In contrast, the proportion of female lecturers from countries such as Italy or Brazil has increased in recent years, but this tendency has been irregular. The year 2020 was excluded from the figure because only 30 lectures were delivered that year, 24 of which were delivered by lecturers from the USA; accordingly, other countries contributed practically no lecturers during that year.

**Fig 10:**
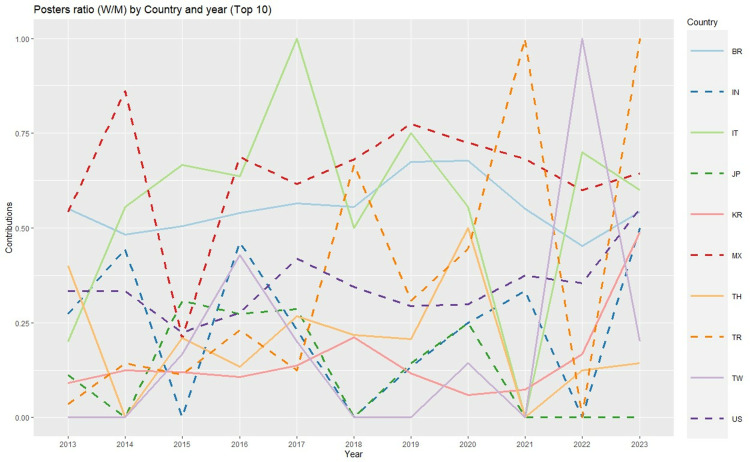
Poster ratios (women/men) by country and year [Top 10 countries] (Abbreviations: AU: Australia, BR: Brazil, CA: Canada, CN: China, DE: Germany, IT: Italy, JP: Japan, KR: South Korea, TW: Taiwan, US: United States of America).

Among the top 10 countries in terms of posters (Fig 10) (without representation of the year 2020 for the same reason as mentioned previously), two very productive countries, i.e., Brazil and, to a greater extent, Mexico, tend exhibit percentages of women that reach approximately 50% or even higher. This research highlights a notable increase in the participation of women from South Korea and the USA in recent years, although South Korea exhibited a fairly small percentage of women in between 2018 and 2021. Additionally, Italy features a good but irregular proportion of women over time, particularly with respect to 2021.

### Thematic analysis

**Fig 11:**
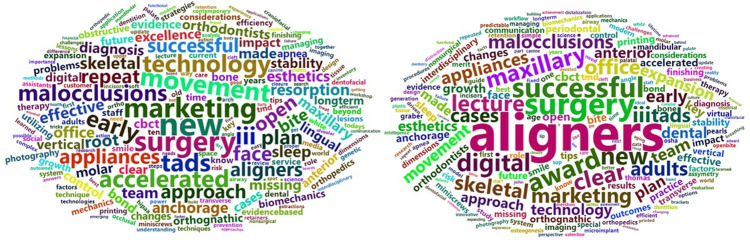
Thematic clouds for lectures. Periods 2013-17 (left) and 2018-23 (right).

**Fig 12:**
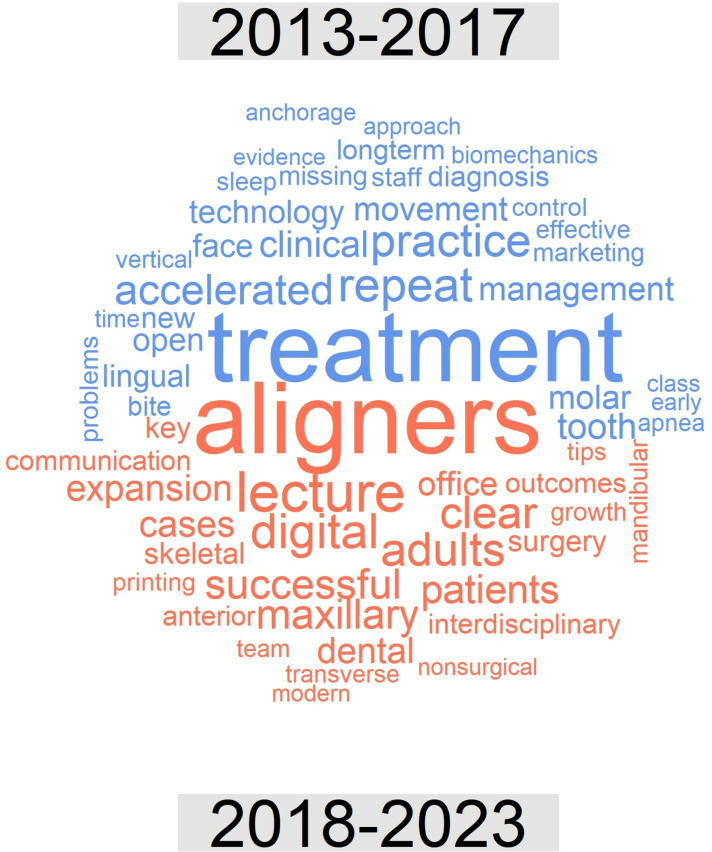
Comparison cloud for lectures indicating the words that appeared most frequently in each period (2013-2017, top, blue colour; 2018-2023, bottom, red colour).

With regard to *topic*, in the thematic cloud associated with the titles of the lectures presented from 2013 to 2023, before the exclusion of very common or nonspecific terms, the most frequent word was “treatment”. The thematic clouds for the periods 2013–2017 and 2018–2023 after the exclusion of these terms are illustrated in Fig 11. Fig 12 presents a comparison cloud that indicates which words appeared more in one period than in another, in which context the increased presence of the terms aligners and digital in the final period was especially significant.

**Fig 13:**
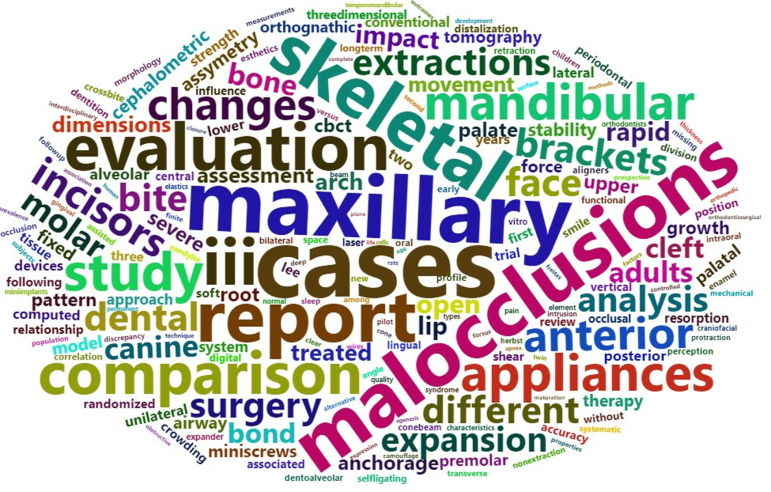
Thematic cloud for posters [2013-23].

**Fig 14:**
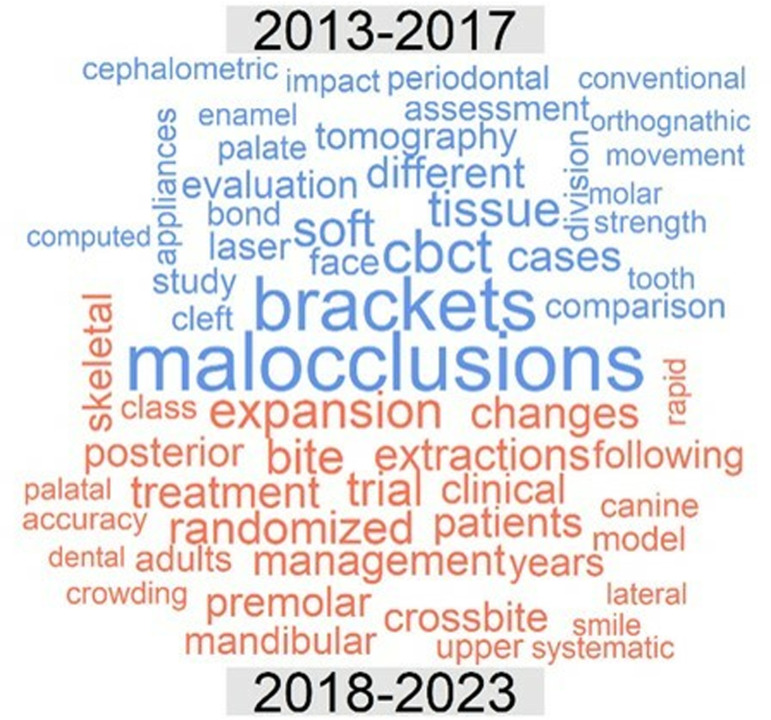
Comparison cloud for posters indicating the words that appeared most frequently in each period (2013-2017, top, blue colour; 2018-2023, bottom, red colour).

**Fig 15:**
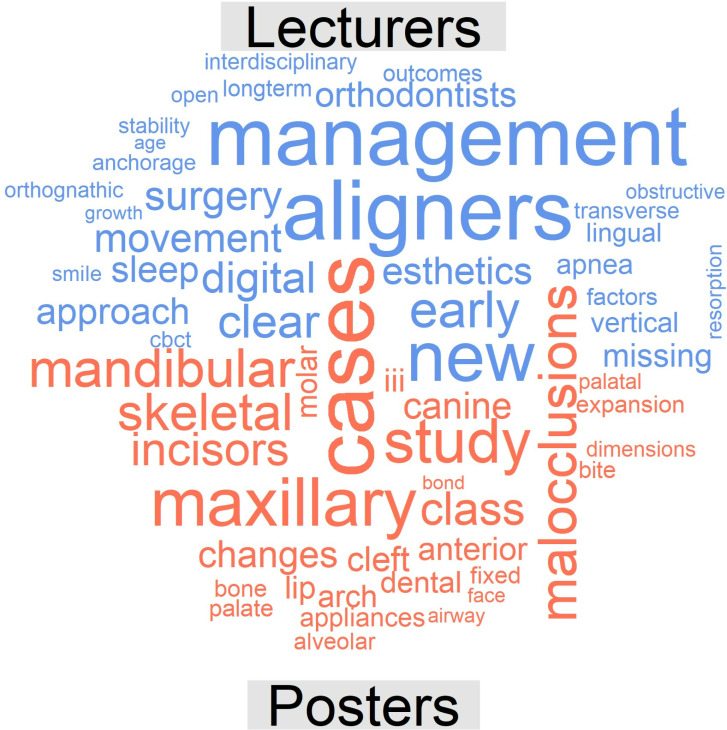
Comparison cloud indicating the words that appeared most frequently with respect to each type of contribution (lectures, top, blue colour; posters, bottom, red colour) [2013-2023].

Fig 13 illustrates the thematic cloud associated with posters after the exclusion of the mentioned terms for the whole period 2013–2023, and Fig 14 and 15 present a comparison cloud between the poster periods 2013–2017 and 2018–2023 and the terms used by lecturers and posters, respectively. The terminology used in the posters seems to be more closely related to studies (cases, studies, reports, evaluations, comparisons, changes, analyses, and assessments), whereas the lectures emphasize other words, such as management and clinical or aligners, treatment, digital, new and aesthetics.

**Fig 16:**
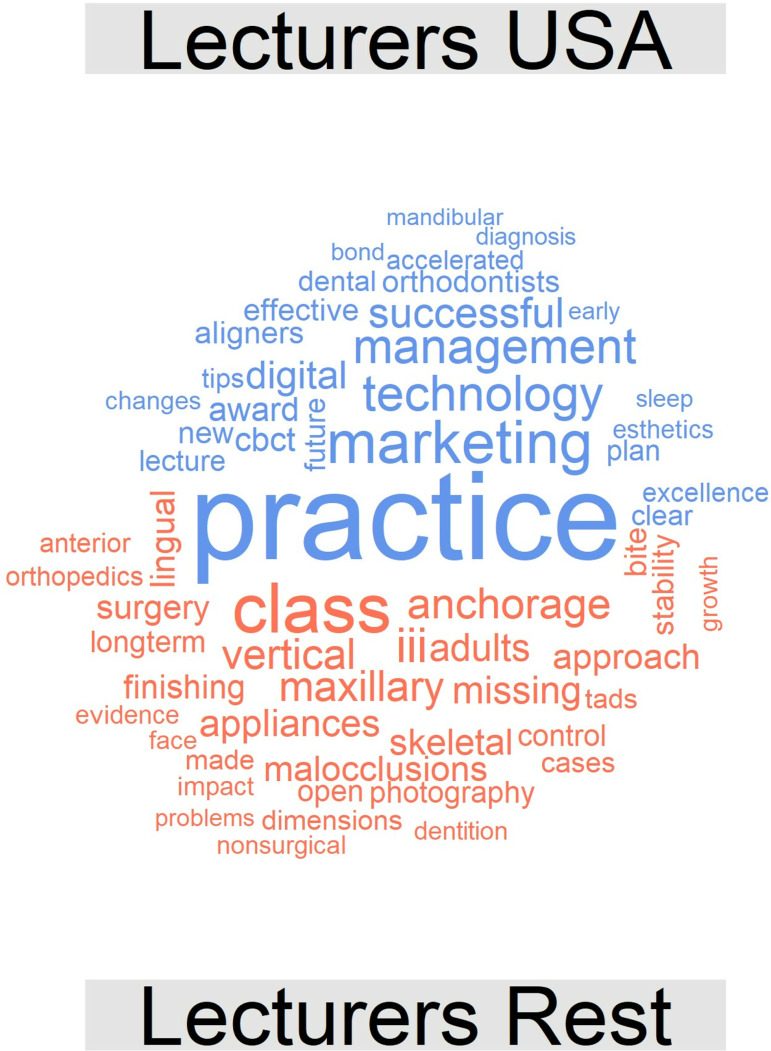
Comparison cloud indicating the words that appeared most frequently with regard to each type of lecturer (lecturers from the USA, top, blue colour; lecturers not from the USA, bottom, red colour) [2013-2023].

Finally, Fig 16 illustrates the comparison cloud between lectures from the USA and those from other countries, which indicates that lecturers from the USA use terms such as practice, marketing, technology, management, patients, success, aligners, new or cone-beam computed tomography (CBCT) more frequently; this result is in line with our subjective and personal experience.

## Discussion

In this study, we analysed the evolution of scientific presentations in orthodontics in light of all presentations made at the AAO during the period 2013–2023. Our study reveals significant changes in the trends of international scientific orthodontic contributions in various respects, such as the nationality of the authors, the immediate effect of the COVID-19 pandemic on these contributions and the corresponding interruptions of certain previous trends, increasing contributions on the part of women, and the development of emerging techniques and themes that clearly highlight the ongoing paradigm shift in orthodontics. The study was also featured an analysis of correlations with different variables that could affect national orthodontic scientific production, some of which were revealed to have a significant influence, as well as, finally, an analysis of the theoretical and real efficiency of contributions by country, which revealed that new countries could make particularly significant contributions to orthodontics at the clinical or research level.

The influence of the AAO extends far beyond its national borders. Due to the historical relevance of this association and the international origins of its members and the participants in its annual meetings, the AAO is one of the most relevant orthodontic institutions worldwide. We analysed orthodontic output from the AAO annual meetings, but scientific contributions to the AAO in general represent a significant indicator of trends in the field of orthodontics.

With regard to *country*, scientific participation exhibits a logical geographical bias since the AAO is a national congress. This bias is mainly evident in the lectures, nearly 70% of which are from the organizing country. The genuinely international character of the annual meetings of the AAO is evident mainly in the presentations of posters, of which the USA accounts for fewer than 20% and is broadly overtaken by Brazil, which is the most productive country. The high numbers of posters contributed by very distant countries such as South Korea (which accounted for more than 10% of the posters) or Turkey (6.33%) is relevant. Importantly, countries such as Mexico, Turkey, Thailand or India, which were not included in the list of the top ten countries producing lecturers, are more visible and make more contributions through presentations in poster format, thus highlighting their high clinical and research potential.

Participation in the annual sessions of the AAO by country is largely in line with the countries that published the most articles in the journals Am J Orthod Dentofac Orthop, Angle Orthod, and Eur J Orthod between 2012 and 2021 [21]. Thus, the USA also produced the most such articles, followed by Brazil. South Korea, Turkey, Italy and Japan also occupy similarly prominent positions in this context. According to the results of our research, Thailand, India, Canada and particularly Mexico are overrepresented in the AAO, whereas the UK, China and Switzerland are underrepresented. Gao et al. [29] highlighted a similar order to that reported by Li et al. [21] during the 2012–2021 period with respect to the same three major orthodontic journals (i.e., the USA, Brazil, South Korea, China, Turkey, the UK, Italy, Japan, Switzerland and Canada) and highlighted the contributions of China and Switzerland in this context, which seem to be particularly underrepresented in terms of AAO presentations. Almotairy [23] conducted an analysis of orthodontic articles that were published between 2011 and 2020 and contained in the Scopus database and identified the USA-Brazil-South Korea triad as the most prolific contributors (in decreasing order); this author also reported that Mexico was overrepresented due to its presence in the top 10, while the UK and Germany were underrepresented.

To identify the factors that may determine the total scientific production of each country, we analysed the seven national indicators mentioned above. Three significant relationships were revealed: both researchers/million and research spending were related to the number of male lecturers (and similar relationships with respect to the number of total lectures were found to be nearly significant), and the distance between the focal country and the USA was negatively related to the number of female lecturers. From these relationships, it is therefore assumed that increased research spending by countries also increases the number of contributions that individuals from those countries make as lecturers; furthermore, increased distance from the USA makes it difficult for women to serve as lecturers.

However, more relationships with the lecture/poster and poster/lecture ratios of scientific contributions seem to be evident. Significant relationships are observed among the ratios of lecturers in a given country, GDP and the variables associated with research (i.e., researchers/million and research spending). This finding indicates that countries in which GDP is higher and those that spend more on research account for a higher percentage of lecture-based contributions than of poster-based contributions.

On the other hand, some of the national variables included in this analysis do not seem to have strong relationships with the total scientific output of every country at the AAO annual meetings. Therefore, it is notable that the number of dentists is not related to any variable, thus highlighting the importance of identifying other indicators associated with dentistry that may be more relevant. Furthermore, the relationships identified in this research are related to the number of lectures, but the number of posters does not seem to exhibit such relationships, thus highlighting the heterogeneity associated with this type of contribution in terms of being listed among the top 10 countries of all types at the economic and population levels. The number of inhabitants is also not observed to be related to scientific contributions, thus identifying research spending and economic level as the most significant factors with respect to scientific production at annual AAO meetings.

The low correlations observed in this research indicate the existence of variables that we did not assess, which may exhibit stronger relationships with the number of contributions made to the AAO annual sessions. This possibility encouraged us to rule out a regression analysis, since this type of analysis assumes that the independent variables are capable of explaining the dependent variable to a large extent, which cannot be guaranteed in our case. For this reason, we additionally conducted an efficiency analysis, which was based on our “Weighted Scientific Production Index”; in this analysis, Brazil was particularly significant, with an advance of 12 positions in terms of lectures and was even the leading country in terms of posters. Future studies should investigate whether these highly efficient countries (i.e., Brazil, Italy, India, Mexico, Turkey and Thailand) could be developing especially significant clinical or research contributions to orthodontics. In fact, Italy featured the highest number of article citations, and Brazil and Turkey occupied the second and fifth positions in terms of the number of articles published in the Am J Orthod Dentofac Orthop, Angle Orthod, and Eur J Orthod [21].

With respect to *year*, the effects of the COVID-19 pandemic are worth highlighting; this event obviously strongly impacted the 2020 annual meeting, as in the case of most scientific disciplines, albeit to different degrees [30]. The pandemic could have been a determinant of changes in long-term trends in some respects. Thus, initially, in 2020, the number of lectures was greatly reduced when the annual meeting shifted to an online format, whereas the number of posters not only did not decrease but even increased. Since 2021, the number of posters has decreased significantly, and a nearly stable reduction was observed during subsequent meetings, whereas the number of lectures nearly recovered to its normal level by the 2023 meeting, leading to very similar numbers of posters and lectures. Whether this change in terms of poster presentations was circumstantial (i.e., associated with the reduced participation of distant countries such as Brazil, South Korea or Turkey, which could be concerned about their countries’ health policies or and the cancellation of their flights—or even the conference itself—for health reasons) or structural can be determined only over time, but the truth is that Brazil’s contributions in terms of posters have been reduced since 2013, and the pandemic negatively impacted this country’s leadership, leading it to be surpassed by the USA in the past two years.

With regard to *gender,* a clear imbalance in favour of men persists, a finding that is generally reflected in scientific presentations at the general, dental and orthodontic levels. Our analysis of this topic reveals both lights and shadows. The most negative point in this regard pertains to the significant imbalance observed in the number of lecturers (3:1), which is not as evident with respect to the posters; this finding seems to indicate that this gender imbalance is exacerbated with respect to opinion and research leaders. Furthermore, the USA, the country that contributes the overwhelming majority of lecturers, has exhibited similarly low proportions of women over the years.

This same finding has been unequivocally related to the speakers invited to the European Orthodontic Society 2015–20 annual conferences [10] or the 2018–19 dental conferences held in the UK [31], particularly in the fields of orthodontics and periodontics, and it has been extended to encompass the directors of different dental societies [16,17]. However, the results regarding the AAO in terms of gender exhibit a stark contrast with the authorship of articles in eight orthodontic journals included in the Journal Citation Report (JCR), in which context the proportion of women was already 39% in the period 2007–17 [5]. In this most recent study, the authors reported a major difference between North America and the European (non-European Union) group, which exhibited a high percentage of female authors in 2017 (60%), thus representing a stark contrast to North America (30%). This finding highlights the need to reinforce the active gender equality policy for lecturers at the AAO annual sessions to overcome the so-called “glass ceiling effect”.

Several positive points are also worth noting. Namely, it is very relevant that 2023 represented the first year in which women presented more posters than did men, although this situation could have been the result of a rebound effect given that the COVID-19 pandemic affected the presentation of posters by women to a greater extent, as in other scientific fields, particularly among women with young children [30]. This inversion in favour of women could soon lead to a greater selection of female lecturers than is currently the case; namely, the ratio of lecturers is already 1:1 in certain countries (Brazil and Italy). Furthermore, the upwards trend observed in recent years with regard to the presentation of posters by women includes countries that feature very high rates of scientific participation in the annual sessions (Brazil, Mexico, the USA, South Korea, and Italy). The AAO itself is in favour of this balance and promotes active leadership policies that reflect the diversity of the association’s membership. Thus, the AAO features a Special Committee on Women Orthodontists whose goal is to support and inspire women orthodontists by helping them develop tools and pathways to leadership, thus ensuring that women serve as AAO trustees, delegates and council members, although we believe that only a multilevel approach that includes a more inclusive media image and that takes into account more than the female component of the community can avoid what Grogan [14] calls the “leaky pipeline” of women in STEMM, which could affect other minorities as well.

In terms of *topics*, the quantitative analysis conducted for this research allows us to identify the significant changes that have occurred in orthodontics in recent years. Thus, certain significant terms have emerged in lectures, including “clear/aligner” (in relation to the development of orthodontic treatment based on transparent aligners), “digital” (in relation to the large number of new digital diagnostic and therapeutic developments), “maxillary”, “adults” and “expansion”. Some of these terms were also included among the most frequent keywords in the most recent articles published by the journals Am J Orthod Dentofac Orthop, Angle Orthod, and Eur J Orthod between 2012 and 2021 [21]; these terms include “Invisalign”, “maxillary expansion” and, to some extent, “cone-beam computed tomography (CBCT)”. Similar considerations were reported by Adobes Martin et al. [22] regarding the terms CBCT and clear aligner but not maxillary expansion based on an expanded analysis of the years 2009–2018 with regard to the dentistry, oral surgery, and medicine (DOSM) category of the JCR.

Other terms whose presence in this context increased, such as “interdisciplinary”, “skeletal”, “transverse”, “surgery”, “nonsurgical” or “team”, as well as the appearance in the cloud of the term “printing” (in reference to 3D printing) clearly define the clinical changes occurring in orthodontics and are very representative of the current state of orthodontics. We did not observe any relevant emergence of the keywords “tooth borne”, “functional appliance” or “oral health” in our final period, unlike Li et al. [21]; this discrepancy may be the result of methodological and date-of-interval differences. “Maxillary” and “canine” were especially prevalent among posters in our final period, similar to the findings reported by Li et al. [21].

Among the decreasing trends observed in the context of lectures, the term “treatment” is worth highlighting, as although this term remains the most frequent. A decrease in the use of the term “accelerated” (in reference to accelerated orthodontics) is also notable; this topic reached its peak 8–10 years ago, but current interest in this theme seems to be lower. Other terms for lectures that exhibited such a decreasing trend included “practice”, “management”, “clinical”, “lingual”, “sleep”, “anchorage”, “missing”, “apnea” and “early”, which are thus revealed to be less active topics in contemporary orthodontics.

The decreased presence of two terms that were a primary focus of orthodontics throughout the 20th century, i.e., “brackets” and “cephalometrics,” in posters was no less significant. The decreased use of these terms indicates the current state of orthodontics. This finding could be viewed as similar to the decrease in publications pertaining to “adhesion” or “friction” in the first decade of this century [22].

Likewise, it is important to appreciate the fact that lecturers from the USA include terms that differentiate them from the rest of the world and emphasize the themes of “practice”, “technology”, “management”, “patients”, “success”, “aligners” or “CBCT”, a result that is in line with our subjective and personal experience. It is possible that lecturers from the USA are more focused on the clinical management of patients, and the advanced research associated with these lecturers increasing their likelihood of lecturing on “new technology”, “aligners”, and “digital procedures”.

Finally, several limitations of this study should be mentioned. The affiliation of authors entailed certain difficulties in some cases. It is sometimes difficult to assign a country to an author in an increasingly international world; thus, we focused on the country of the institution of the first author. Similarly, in some cases, our classification of authors in the framework of a binary regime (woman/man) might not be appropriate. Data concerning the authors’ ages could have enriched our analysis of gender differences in scientific presentations and clarified whether the greater presence of men is associated with seniority, especially in the case of lecturers. Information regarding the number of rejected lectures or posters was not provided and could not be determined. Finally, conducting the analysis over a longer period of time would perhaps have enhanced our ability to detect changing trends.

It is important to reflect on these findings and seek opportunities to improve participation and representation in scientific events, thereby promoting diversity in terms of nationality and gender. Additionally, the lessons learned during the pandemic can be useful with regard to the need to strengthen the virtual aspects of conferences and facilitate remote collaboration. This approach could offer new opportunities to expand global participation and the dissemination of scientific research beyond geographical barriers.

## Conclusions

The nationalities of the lecturers, who were mostly from the USA, are not closely related to those of the posters, particularly with regard to the USA, Brazil, Canada, Mexico and Turkey. Research spending and the economic level (GDP) are the most important factors determining the type and number of a country’s contributions. In countries in which GDP is higher and those that spend more on research, a higher percentage of contributions take the form of lectures rather than posters.

In terms of gender, a clear imbalance in favour of men persists among lectures. Increased distance from the USA makes it more difficult for women to serve as lecturers.

The format of the presentations seems to condition their content in part, and differences in topics were observed between lectures and posters. Thematic analysis reveals an emergent paradigm shift concerning topics of current interest in the field of orthodontics towards the terms clear/aligners and digital in the context of lectures. The topics that were most commonly referenced by lecturers from the USA differ from those referenced by lecturers from other countries.

## Supporting information

S1 Appendix Sample Data. (XLSX)
